# Supplementary material for: Are carbon emissions trading and green financial instruments synergistic? -Comprehensive quantitative research based on content analysis
Source: PLoS One. 2024 Mar 7;19(3):e0298601. doi: 10.1371/journal.pone.0298601 (PMC10919601; doi:10.1371/journal.pone.0298601)
Supplement: S1 Data — (ZIP) [file pone.0298601.s001.zip › Data/地级市Z_F工作报告.zip!/╒π╜¡/╬┬╓▌╩╨/└┤╫╘╣1⁄2╓┌║┼í╛┬φ┐╦╩2╛▌═°í┐_2014.docx]

各位代表：

　　现在，我代表温州市人民政府，向大会作工作报告，请予审议，并请市政协委员和其他列席人员提出意见。

一、2013年主要工作

　　2013年，是温州发展极其困难的一年。我们在省委、省政府和市委的正确领导下，牢牢把握稳中求进工作总基调，着力实施“十大举措”，奋力推进赶超发展，全市经济企稳回升，社会和谐稳定，各项事业取得新进展。实现地区生产总值4004亿元，同比增长7.7%；公共财政预算收入324亿元，增长11.9%；全社会固定资产投资2950亿元（其中限上固定资产投资2618亿元），增长25.1%；社会消费品零售总额2136亿元，增长11.2%；外贸出口总额181亿美元，增长2.6%；城镇居民人均可支配收入37 852元，农村居民人均纯收入16 194元，分别增长8.7%、10%；居民消费价格指数上涨1.9%；节能减排超额完成省下达任务。

　　（一）着力振兴实体经济，稳定增长和转型升级取得实效。推出系列刺激经济举措，工业生产、限上消费、外贸出口等主要指标逐步回升。实现工业增加值1768亿元，增长7.6%；规上工业企业利润增长9%。建筑业产值突破千亿元。第三产业增势良好，网络零售额增长108.3%，旅游总收入增长20.2%。农业生产保持稳定，建成粮食生产功能区22万亩、现代农业园区99个。深入实施创新驱动发展战略，推出“人才房”政策，新引进北航温州研究院等研发机构16家、国（境）外专家项目154项，入选“国千”人才7名、“省千”人才18名。研究与试验发展经费支出占生产总值比例1.4%。科技进步综合评价从全省末位跃至第五位，荣获全国科技进步先进市称号。深化国家知识产权、质量强市示范城市建设，专利授权量增长39.4%，鹿城创成首批国家知识产权强县工程示范区，国家汽车电气零部件、阀门质检中心分别落户瑞安和永嘉。大力实施“三转一市”“四换三名”，完成“个转企”2.3万家、“小升规”1471家、“规改股”134家，新增高新技术企业72家，技改投资增长37.6%，新产品产值增长46.2%；关停重污染、高耗能企业396家，占全省三分之一多。

　　（二）着力扩大有效投资，发展后劲和保障能力不断增强。交通、能源、水利等基础设施建设提速推进，绕城高速西南线、市域铁路S1线、金温铁路扩能改造等工程进度加快，甬台温高速复线南塘至黄华段开工，龙丽温（泰）高速作为省重点支持项目取得突破性进展，龙湾国际机场新跑道投用，乐清湾港区一期码头主体工程完工，诸永高速延伸线（瓯江过江通道）主桥贯通，雁楠公路、滨海大道建成通车，华润苍南电厂一期并网发电，丽水36-1气田陆域终端基本完工，西向排洪主体工程投用。瓯飞一期、瓯江口浅滩二期等围垦项目加快推进，在围面积19.3万亩。浙南沿海先进装备产业集聚区获批，温州经济技术开发区整合提升方案通过省政府评审，瓯江口新区建设步伐加快，小微企业园建设全面启动。完成工业投资606亿元，增长29%。实施“三招一优”，推动温商组团回归，实际到位内资410亿元，增长94%；实际利用外资5亿美元，增长25.9%。列入国家城镇低效用地再开发试点，新增建设用地3.8万亩，到位建设用海指标1.16万亩，清理转而未供、供而未用土地6.8万亩。

　　（三）着力加强城乡建设，功能提升和环境再造成效明显。城市总体规划修改基本完成，组团城市建设整体推进，龙湾、瓯海中心区和各县（市）新区开发全面提速，瓯洞一体化格局初步构建。小城市培育和中心镇建设有力推进。城市单元更新有序推开，市区“三大亮点区块”启动实施，滨江商务区30幢大楼加快建设，“印象南塘”初展形象。“三改一拆”工作走在全省前列，累计改造城中村、旧住宅区、旧厂房3253万平方米，拆除违法建筑2565万平方米。“四边三化”和绿化建设成效明显，绿化造林15.2万亩，建成森林公园42个、滨水公园119个、绿道1980公里，市区人均公园绿地面积新增5平方米。获得省级园林城市、省级森林城市称号，国家低碳试点城市获批，泰顺创成国家级生态县，洞头入选首批国家级海洋生态文明示范区。实施国家级“智慧城市”试点，成为全国首批移动4G正式商用城市。美丽浙南水乡建设拉开序幕。完成温瑞塘河沿河10个片区污水管网整治，垃圾河、黑臭河治理取得进展。提前3年完成珊溪水源地畜禽整治，库区干部群众作出了突出贡献。扎实推进美丽乡村建设，完成392个待整治村整治，建成农村联网公路262公里。实现镇镇电气化。

　　（四）着力深化改革开放，改革试点和开放合作取得突破。“五大国家级改革试点”扎实推进。《温州市民间融资管理条例》通过省人大立法，“温州指数”信息采集拓展到全国31个地市。中长期贷款增长27.5%，直接融资增长59.4%。地方金融监管体系初步构建。人民银行征信中心温州分中心挂牌设立，市企业公共信用信息平台覆盖35个部门、22.7万家企业。村股份经济合作社清产核资基本完成，新设立农村资金互助会23家，新增土地流转面积24.8万亩。第二批300所民办学校改革试点全面展开。19个社会力量办医项目可新增床位7000张。创新社会组织登记管理，启动社会组织承接政府职能转移试点。口岸开放成效显著，台湾居民“落地签”、通用航空基地获批。开通温州至罗马国际航线，成为国内首个直飞欧洲的地级市。浙台（苍南）经贸合作区建设取得实效，成功承办“浙江·台湾合作周”活动，开启对台合作新局面。圆满完成援藏、援疆、援青三年任务。

　　（五）着力保障改善民生，社会事业和社会管理持续进步。全市公共财政用于民生支出330.8亿元，增长11.8%，年度十大民生工程全面完成。市区安置房交钥匙4480套，完成“两证”办理16 924个，“安置提速”三年任务已完成86%。建成市区主干道路39条，开发地下空间358万平方米，新增停车泊位2.4万个、公交专用道30.8公里，新增、更新公交车223辆，公共自行车实现“一城一网、通借通还”，公交分担率达25%。基本完成学前教育三年行动计划，成功启动市区初高中分设办学。中职教育整合提升和高职院校创办分院加速推进。温州医学院更名为温州医科大学，温州肯恩大学去筹通过评估，温州市委党校、温州城市大学新校园启用，浙江安防职业技术学院获准筹建。成功举办首届市民文化节和第十五届市运会，建成文化礼堂309个，一批专题博物馆向市民开放，多人多项文艺作品获国家级最高奖，温籍健儿在第十二届全运会上取得佳绩。公立医院改革稳步开展，国家基本药物制度全面实施。基层卫生服务体系逐步完善。人口计生工作不断加强，出生人口性别比下降4.5个比值。加大社保扩面、低保调标力度，城乡居民医保实现一体化。农村社区居家养老服务照料中心覆盖率达75%。强化食品加工流通消费环节整治，食品药品安全保障得到加强。创新基层“网格化”管理模式，联动推进消防安全、安全生产、居住房屋出租管理和新居民服务管理。大力实施绿色生态殡葬改革。加强应急体系建设。成功防御“菲特”强台风，灾后重建有序推进。积极构建立体化社会治安防控体系，严厉打击各类违法犯罪活动，大力推进历史遗留问题和信访积案化解，平安大市“五连创”扎实开展。工青妇、老龄、残疾人、慈善等事业全面发展，民族、宗教、外事、侨务、双拥、扶贫等工作取得新成绩。文成、泰顺圆满完成省政府“三年特扶”任务。

　　（六）着力优化政府服务，发展环境和自身建设得到加强。开展“万名干部进万企解万难”行动，2.2万名驻企服务员、金融服务员深入企业破解难题。坚持“政银企法”联动，强力推进风险企业帮扶和不良贷款化解，政府应急转贷金循环使用累计超450亿元。落实结构性减税、社保缓缴减征等政策，减轻企业负担近60亿元。系统推进行政审批、中介机构和招投标制度改革，市本级设定的非行政许可审批事项全部取消，建设项目审批时限压缩60%，中介服务提速44%。主动接受人大依法监督和政协民主监督，办理人大代表建议459件、政协提案447件，推动解决了一大批经济社会发展问题。完善法治政府建设考核评价体系，细化量化行政裁量权，落实行政规范性文件“三统一”制度。完成市级机关事业单位在编不在岗人员清理。开展“万人评议机关中层和基层站所”活动，对31种损害发展环境的行为实行问责。严格落实中央“八项规定”、省委“六个严禁”，市本级“三公”经费支出下降21%。强化审计监督和行政监察，廉政建设进一步加强。

　　各位代表，过去一年我们经受了严峻考验，取得了来之不易的成绩，这是全市上下和衷共济、克难奋进的结果。在此，我代表市人民政府，向在各个领域辛勤工作、作出贡献的全市人民，向给予政府工作大力支持的人大代表、政协委员，向所有参与、关心、支持温州建设发展的同志们、海内外朋友们，致以崇高的敬意和衷心的感谢！

　　我们也清醒地看到，我市经济社会发展中还存在不少困难和问题。经济回升的制约因素还很多，增长动力不够强，出口、消费等指标未达到预期目标。经济发展中的结构性问题和深层次矛盾依然存在，产业“低小散”格局没有根本改变。局部金融风暴影响尚未消退，银行不良贷款处置和企业互保链化解任务艰巨，金融生态、信用体系亟待修复。城乡规划建设管理不够精细，中心城市集聚辐射能力有待提高，城乡区域发展不平衡、不协调。社会建设和管理存在不少薄弱环节，生态环境、市容卫生问题突出，消防、安全生产形势严峻，改善民生与维护社会和谐稳定任务繁重。政府职能转变仍然滞后，部分政府工作人员的责任意识、担当精神、工作作风与赶超发展的要求不相适应。对此，我们一定高度重视，采取有效措施，认真加以解决。

　　二、2014年工作总体要求和目标任务

　　2014年，是全面深化改革的开局之年，也是推动赶超发展的关键之年。按照市委十一届四次、五次、六次全会部署，今年政府工作的总体要求是：认真贯彻党的十八大和十八届二中、三中全会，及省委十三届四次全会精神，深入实施“八八战略”，按照“干好一三五、实现四翻番”的要求，坚持和发展“温州模式”，围绕“三生融合·幸福温州”战略目标，以改革创新统领全局，全面落实“十大举措”，切实抓好“三十项发展重点”和“四十项改革项目”，力争今年经济增速跟上全省发展步伐，力争重点领域和关键环节改革走在全省乃至全国前列，确保社会和谐稳定，努力开创温州赶超发展新局面。

　　在工作中，我们将坚守三条“底线”，构建四大优势。把百姓增收、生态良好、社会平安作为经济社会发展的“底线”，作为政府工作的“标尺”。着力重构体制机制优势，使市场在资源配置中起决定性作用，充分激发民间活力；着力培育产业竞争优势，加快发展特色分明、链条配套的现代产业集群，重振实体经济雄风；着力打造区位条件优势，加速温州大都市区崛起，增强中心城市集聚辐射能力；着力发挥温州人资源优势，把温商作为第一资源，把温州人创业创新精神作为最可宝贵的财富，为推动温州赶超发展注入不竭动力。

　　建议2014年全市经济社会发展的主要预期目标为：地区生产总值增长8%左右；公共财政预算收入增长8%；限上固定资产投资增长15%以上；社会消费品零售总额增长12%；外贸出口增长5%；城镇居民人均可支配收入和农村居民人均纯收入分别增长8.5%、9.5%；研究与试验发展经费支出占生产总值比例1.6%；节能减排完成省下达的任务。

　　根据上述目标要求，今年政府工作着重要在抓改革、稳增长、促转型、强统筹、治环境、惠民生等方面取得积极成效。

　　（一）加大改革突破力度，增强发展内生动力

　　以更大的魄力推进政府自身改革。建立政府权力清单制度，试行企业投资负面清单管理模式，开展负面清单外企业投资项目政府不再审批试点。继续取消、下放一批市级审批事项，推进市县两级同权扁平化审批试点。开展审批流程再造，建立全流程公开审批机制，推动“事前审批”向“事后监管”转变。培育和规范中介服务机构，审批中介服务机构与主管部门、挂靠单位彻底脱钩。整顿规范招投标市场。开展新一轮政府机构改革，科学设置政府部门职能，严格控编控人控机构。推进生产经营类事业单位改制。深化公共财政体制改革，扩大部门预决算公开。健全政府性债务预警管理机制。推进瓯洞一体化行政管理体制改革，力争撤县设区获批。开展功能区管理体制改革试点。在县（市）推广实施相对集中行政处罚权工作，探索基层综合执法，不断提高中心镇社会治理能力。

　　健全完善现代市场体系。把发展提升民营经济放在更加突出位置，努力消除各种隐性壁垒，推动民间资本顺畅进入基础设施、基础产业、公共服务等领域。加大要素配置市场化改革力度，建立要素配置评价标准，加强要素交易平台建设，推动用能、股权等进场交易。坚持“亩产论英雄”，健全完善土地公开公平供应机制、低效利用土地市场化退出机制，实行差别化地价、电价、水价征收政策。推进碳排放权和碳汇交易试点。用好“海域直通车”政策，探索建立海域海岛收储制度。深化国资体制改革，建立现代企业制度，发展混合所有制经济。做好我市第三次全国经济普查工作。坚持政府以身作则，以政务诚信示范引领全社会诚信建设，争创全国社会信用体系建设示范城市。

　　全面深化“五大国家级改革试点”。深化金融综合改革。着力完善资本市场体系，支持企业通过多层次资本市场扩大直接融资，探索开展小额贷款公司发行优先股试点。实施普惠金融工程，抓好小微金融服务中心建设，探索发展互联网金融。鼓励金融机构创新推出符合市场需求的利率市场化产品。构建社区银行体系，筹建民营银行，引进证券、保险和政策性金融机构，完成农村中小金融机构股改，丰富地方金融业态。组织实施《温州市民间融资管理条例》，激活民间融资。规范民间资本管理公司发展，促进民间资本与实体经济有效对接。深化农村综合改革。规范村经济合作社运行机制，抓好农户承包地、农民宅基地（住房）、村集体建设用地“三确权”，开展农民住房财产权抵押、担保、转让试点。建立健全市、县、乡三级农村产权交易平台，推进涉农产权有序流转交易。实施国有林场改革。规范提升农村资金互助会。扩大农业政策性保障范围，提高农业、农民抗风险能力。积极稳妥开展户籍制度等配套改革，有序推进农业转移人口市民化。深化民办教育综合改革。编制民办教育发展规划，落实民办教育改革政策，推动试点学校规范提升发展，努力把“全国试点”办成“全国示范”。深化社会力量办医综合改革。鼓励民间资本参与公立医院改制重组、投向健康服务等领域，加大高端优质医疗资源引进力度，加快发展医养结合社会关怀机构。深化民政综合改革。按照“养事不养人”思路，加大政府购买服务力度，积极推开社会组织承接政府职能转移试点。加强基层组织建设，完善提升社区公共服务功能。加强专业化社工队伍建设。

　　（二）坚持“三轮驱动”，确保经济持续稳定发展

　　发挥投资的关键作用。启动实施“温州赶超发展三年超万亿投资计划”，促进投资稳定增长和结构优化，今年限上固定资产投资突破3000亿元，其中工业投资比重26%以上，民间投资比重62%。强化“三招一优”，坚持引温商、招央企、选外资三管齐下，实施“温商回归三年超三千亿工程”，推进“百名领头雁工程”，组织在外商会组团回乡投资考察，力争全年到位内资800亿元。加强重大项目前期工作，着力谋划一批大项目、好项目。完善领导干部联系重大项目制度，深入开展“拔钉清障”专项行动，坚决扫除影响项目落地建设的一切障碍。探索发行“蓝海股份”，加强用海报批，积极推进“三年实施20万亩围垦工程”。用好国家城镇低效用地再开发试点和荒滩未利用地开发试点政策，科学开发利用低丘缓坡，盘活低效土地2.1万亩。完成垦造耕地2万亩，其中围垦垦造耕地1万亩。

　　发挥消费的基础作用。千方百计增加居民收入，提高消费能力。强化政策落地，着力培育文化娱乐、体育休闲、健康养老等新型消费业态，扩大信息、旅游、餐宿等服务消费，促进消费持续增长。完善商贸市场、商业网点发展规划，改造提升五马、大南等传统商圈，有序发展城市综合体，加快培育新兴城市商圈。推进专业市场“迁改并转”，开展新一轮农贸市场改造提升。加快组团城市、重点镇商贸设施建设，提升县城、中心镇商贸业态和消费环境。深化万村千乡市场工程，促进产销对接、农超对接，健全农村现代流通网络。办好2014国际轻工暨时尚消费博览会、温州进口商品展等一批重点展会，繁荣商业氛围。

　　发挥出口的支撑作用。稳定政策预期，加大扶持力度，加强与中信保的战略合作，加快中国鞋类出口基地和6个省级出口基地建设，着力扩大外贸出口。配套落实国家有关政策，鼓励企业扩大先进装备、关键技术和原材料进口，积极打造国际时尚消费品集散基地。探索个人境外直接投资试点。提升发展境外工业园和专业市场，支持有条件的企业并购境外企业、研发机构和知名品牌。加快海港、空港口岸国际化步伐，确保状元岙港区扩大开放获国务院批准，乐清湾港区水陆域开放获批。积极申报综合保税区，争取保税物流中心（B型）获批。启动电子口岸建设，推行关检合作机制，逐步实现“一次申报、一次查验、一次放行”，提高通关效率和贸易便利化水平。加强与国际友城、港澳台地区的交流合作，争取港澳“自由行”、台湾“个人行”和海峡两岸（温州）民营经济创新发展示范区获批，推进各县（市、区）对台合作特色平台建设，支持苍南申报国家级台商投资区。积极对接上海自贸区，深化区域协作。

　　（三）加快产业转型升级，增强竞争实力

　　实施“五一○产业培育提升工程”。做强做大电气、鞋业、服装、汽摩配、泵阀等五大支柱产业，大力发展网络经济、旅游休闲、现代物流、激光与光电、临港石化、轨道交通、通用航空、新材料、文化创意、生命健康等十大新兴产业。今年力争电气行业产值率先超千亿元。把时尚产业作为温州标志性产业加以重点培育，着力打造“中国时尚消费之都”。重视发展高端电镀、精密模具等基础产业，引导鼓励全产业链发展，推动传统块状经济向现代产业集群转型。

　　全力打造工业强市。突出工业主导地位，开展工业强县、强镇、强企创建，支持乐清深化省级工业强县建设，鼓励瑞安争创省级工业强县。加大“腾笼换鸟”“机器换人”力度，实施“百项百亿技改工程”，技改投资增长30%。推进工业化、信息化深度融合，提升生产智能化、装备自动化水平。深入实施“三名”工程，培育一批龙头骨干企业。提质推进“三转一市”，新增规上企业800家、股份制企业100家，推动50家企业上市和在新三板、区域性股权交易平台挂牌交易。增加工业用地供给，除鹿城、文成、泰顺和温州生态园外，各地要确保年度新增建设用地的40%用于发展工业。强化工业项目开工率、竣工率、投产率考核，开工建设400个以上已供地未开工项目。大力发展建筑工业化，促进建筑行业提质增效。

　　加快发展现代服务业。全域谋划旅游产业发展，加强整体营销，推进重大旅游项目和配套设施建设，深化雁荡山等旅游功能区体制改革，加快雁楠旅游一体化步伐，支持楠溪江、百丈漈、洞头争创5A级景区，泰顺廊桥——氡泉和文成天顶湖申报建设省级旅游度假区。大力培育网络经济“一号新产业”，着力引进知名电商企业，加快建设网络经济集聚区，培训“网商大军”15万人次，推进电子商务向各领域拓展，网络零售额突破500亿元。编制实施新一轮物流业发展规划，建设一批重点物流园区，培育一批龙头物流企业，促进现代物流与制造业、商贸业、电子商务联动发展。抓好文化创意产业园、广告产业园建设，推动工业设计、物联网、信息软件等生产性服务业加快发展。加强总部型经济培育，把本地温商留住，把在外温州人经济引回来。

　　大力发展现代农业。深化“三位一体”农村合作体系建设，规范提升农民合作组织，积极培育家庭农场、种养大户和专业合作社等新型农村经营主体，推进农业产业化、现代化。深化农业“两区”建设，建成粮食生产功能区11万亩、现代农业园区30个。加强粮食储备调节，提高粮食安全保障能力。实施地力提升工程，建成高标准基本农田25万亩。新增农村土地流转面积10万亩。实施“科技兴农”，强化农业技术支持，抓好良种推广。大力发展生态循环农业，积极培育绿色和有机农产品。加强动植物疫病防控，保障农产品质量安全。

　　强化产业平台支撑。全面启动浙南沿海先进装备产业集聚区规划建设，高强度推进瓯江口产业集聚区开发，力争各引进1个超20亿元产业大项目。加快温州经济技术开发区和省级工业园区整合提升，抓好国家级高新区、大学科技园核心区建设，鼓励有条件的县（市、区）和功能区争创省级高新园区，建设一批科技孵化器。围绕“三年建成2400万平方米小微企业园”目标，严把准入门槛，今年启动建设30个有示范作用的高质量小微企业园。培育蓝色经济板块，大力发展海洋新兴产业、临港先进制造业、海洋旅游业、现代海洋渔业，加快推进中石化液化天然气、泰地石化等项目，建成海洋科技创业园。

　　增强科技创新能力。围绕“八倍增、两提高”目标，增加财政科技经费支出，力争各项创新指标增速达到全省平均水平，努力创建国家创新型城市。鼓励企业加大创新投入，深化科技与金融结合试点，新增高新技术企业100家、科技型中小企业500家，培育省级企业技术中心5家、研发中心20家。统筹全市科技资源，办好各类院地合作创新平台，促进科技成果转化。引导中小微企业共建技术联盟，着力攻克一批共性关键技术。加强知识产权运用和保护。实施标准强市、质量强市、品牌强市战略，创成全国质量强市示范城市。把人才作为推动创新发展的首要因素，着力解决人才住房、子女就学等问题，力争引进“国千”人才5名、“省千”人才10名，“海外工程师”20名，培养高技能人才2万名。

　　加大助企发展力度。全面落实振兴实体经济一揽子政策，完善驻企服务员、金融服务员制度，及时帮助企业解决发展难题。实施升级版“双十条”帮扶举措，加大企业资金链、担保链问题处置力度，抓好银行不良贷款“控新化旧”工作。深入开展“治赖”追逃专项行动，严厉打击逃废债行为。强化金融支持，扩大信贷投放，努力缓解中小微企业融资难。积极推进企业整合重组。充分发挥行业协会和商会服务企业发展、推动行业转型的重要作用。重视企业用工问题，促进企业劳动力供需衔接。

　　（四）加快大都市区建设，促进城乡发展一体化

　　强化城市总体规划引导，完善大都市区布局体系，统筹推进新型城市化和新农村建设，促进县域经济向城市经济转型，努力建设生态型、组团型、智慧型大都市区。

　　加快构筑都市基础设施网络。全面推进五大高速公路项目，大力实施甬台温高速复线、绕城高速西南线和北线二期，建成诸永高速延伸线，开工龙丽温（泰）高速瑞安至文成段，全力争取文成至泰顺段获国家审批。加强港城、港区联动，探索建立利益共享投融资体制。深化乐清湾、状元岙、大小门岛三大核心港区开发，开工建设乐清湾港区铁路支线，加快推进77省道洞头延伸线，基本建成大门大桥。加快建设龙湾国际机场T2航站楼、航空总部大楼，开工建设通用航空基地、机场交通枢纽。金温铁路扩能改造完成线下工程并具备铺轨条件。市域铁路S1线建设全线铺开，S2、S3线深化前期工作；开工建设瓯江北口大桥，分段实施104国道改造，加快推进南金公路、大罗山环线和鳌江跨江3座桥等干线交通建设，强化大都市区组团联接。开展油气、电力设施建设攻坚，加快甬台温、金丽温油气管道和县（市、区）天然气输配工程建设，实施市区旧住宅区天然气管道改建，实现丽水36-1气田送气入户；争取开工泰顺交溪流域水电站；建成500千伏玉环电厂二期送出工程，加快建设500千伏温西变、温东至南雁线等项目，着力构建500千伏区域环网，强化大都市区能源保障。

　　加快城市有机更新。围绕“靓丽山水智城”建设，加强城市形态、色彩、景观设计，高标准实施滨江商务区、中央绿轴公园、三垟湿地公园“三大亮点区块”，推进鹿城双屿综合整治。抓好滨江景观廊道、塘河景观廊道建设，加强大罗山保护管理开发，开展主要入城口环境整体改造提升。深化国家级“智慧城市”试点。推进4G商用，市区主要公共场所实现免费WiFi全覆盖。提高“三改一拆”工作科学化水平，开展“无违建县”“无违建镇”创建，强力推进城中村改造，完成城中村改造2.8万户、旧住宅区改造50万平方米、旧厂房改造100万平方米，推进现代化美丽县城建设。深化交通拥堵治理，继续“打卡口、接断路、建环线”，建设市区主干道路15条，畅通城区交通微循环；坚持公交优先，新增公交专用道30公里、自行车专用道74公里、停车泊位1.5万个，启动建设首条BRT快速公交专线，公交分担率提高3个百分点。开发地下空间100万平方米。完善交通信息诱导系统，优化城市交通组织与管理，有效缓解行车难、停车难。加强出租汽车管理，提升出租汽车形象。完成市区地下管线普查，强化市政设施管养，提高城市精细化管理水平。

　　加快美丽乡村建设。深入实施“千百工程”，推进连线成片整治建设，打造一批美丽乡村精品线、精品村。加快城市基础设施和公共服务向农村延伸，强化农村公路养护和安全监管，建成农村联网公路100公里，大中修500公里，桥隧改造维修40座；创建新农村电气化市。加强古建筑和古村落风貌保护，抓好57个省级历史文化重点村落保护利用。完善农房集聚模式，有序引导农户跨区域异地集聚、整村搬迁集聚，优先安排下山搬迁、灾害避险搬迁、农村D级危房改造搬迁。

　　加快欠发达地区发展。开辟“山海协作”新路，完善生态补偿资金稳定增长机制，研究制定文成、泰顺等县“工业飞地”方案和政策。积极争取将文成、泰顺列入全国或全省生态主体功能区试点，努力将两县县城列入省级小城市培育试点。巩固珊溪水源地环境整治成果，加大库区群众转产转业扶持力度。实施“低收入农户收入倍增计划”，拓宽农民就业增收渠道。强化对海岛、偏远山区、革命老区和少数民族地区的扶持。

　　（五）加强生态环境保护整治，加快建设美丽浙南水乡

　　突出水环境综合治理。以攻坚之势推进“五水共治”，分步实施“百项千亿”治水项目，今年完成投资164亿元。坚持治污先行，加大平原河网污染整治力度，严格落实“河长制”，基本消灭垃圾河，有效改善黑臭河水质，建成一批示范河道。强力推进鳌江流域综合整治。开展城乡污水、垃圾处理设施建设大会战，全面推进市区中心片等六大污水处理厂建设，完成温瑞塘河沿河19个片区污水管网整治；加快市区西向生态填埋场、综合材料生态处置中心建设，基本建成永强垃圾焚烧厂二期；实施城镇污水处理设施52个、垃圾处置项目27个；新增农村污水全面治理村500个、扩面改造村600个。加大集中式饮用水源保护力度，强化城镇、农村居民饮用水安全保障。加快推进瓯江、飞云江、鳌江三大独流入海工程，建成平阳顺溪水利枢纽、永嘉县城排涝应急工程，加快永嘉南岸水库等重大水利项目前期工作。扩大清洁生产和中水回用。完成河道生态建设三年行动计划，促进水生态、水景观、水文化有机融合。强化土壤污染治理和修复。加强海域海岛海岸带整治修复保护，联动推进乐清湾、瓯江口等近岸海域污染防治。

　　狠抓大气污染防治。加大雾霾监测预警和治理力度，强化工业废气、汽车尾气、施工扬尘等整治，推进燃煤电厂脱硫脱硝及除尘技术设备改造，抓好垃圾焚烧、秸秆焚烧、餐厨油烟污染治理，淘汰“黄标车”1.5万辆，扩大清洁能源利用，有效削减PM2.5排放，逐步改善空气质量。

　　深化“六城联创”活动。加大全国文明城市和国家卫生城市、国家森林城市、国家环保模范城市创建攻坚力度，加快国家园林城市、国家历史文化名城创建步伐。强化政府主导、群众主体，广泛开展群众性精神文明创建活动，推动创建工作向基层社区、单位企业、千家万户延伸。加强农贸市场、背街小巷、公厕等重点部位卫生整治，做好无证小菜场、废品收购点等场所疏导，优化公共秩序，改善市容环境卫生。深入推进“四边三化”“两无三化”，提升绿化层次，加强后期管养，新增绿化造林面积8.2万亩、城市绿地500公顷以上，完成绿道网和森林公园建设三年总目标。

　　抓好节能减排工作。完善能源消费总量和能耗强度双控制度，禁止新上高耗能、高排放项目。深入开展节能低碳行动，争取国家低碳城市创建达标。强势推进六大重污染、高耗能行业整治，全面完成电镀行业整治入园，加快瑞安、苍南印染园区建设，加大合成革行业整治力度，基本完成造纸、制革、化工行业整治验收。

　　（六）推进社会事业加快发展，切实增进民生福祉

　　把满足人民群众的民生需求作为政府工作的出发点和落脚点，切实加大民生投入，明确任务表、时间表、责任表，认真办好十个方面、20项民生实事。

　　坚持教育优先发展。全面推进素质教育，优化调整教育设施布局，创建等级幼儿园100所、省级义务教育标准化学校100所，县（市、区）全部通过国家义务教育基本均衡评估认定。持续推进市区初高中分设办学。深入实施加强学校体育三年行动计划，切实提高中小学生身体素质。大力发展职业教育，加快建设滨海职教中心。支持温州医科大学发展，办好温州大学和温州肯恩大学，全力推进浙江安防学院（筹）、浙江工贸学院、浙江东方学院、温职院瑞安分院和温医仁济学院、温大瓯江学院等高校、高职新校区建设。推动特殊教育、继续教育等各类教育协调发展。利用信息化手段扩大优质教育资源覆盖面。加强教师队伍建设。抓好校园安全和周边环境整治。

　　深化卫生强市建设。继续推进医药卫生体制改革，全面实施公立医院药品零差率销售，破除“以药补医”，建立现代医院管理制度。加快温医附二医瑶溪新院、市中医院新院区、市七医、市中心血站、温州护士学校等项目建设。实施优质医疗资源“双下沉、双提升”工程，推进县、镇、村（社区）医疗卫生机构一体化管理，90%以上的村（社区）卫生室纳入国家基本药物制度实施范围。建设流动社区卫生服务站100个。定向培养农村社区医生200名，培训基层卫技人员1万人次。扶持中医药事业发展。加强传染病和慢性病防控。完善医疗纠纷调处机制，构建和谐医患关系。理顺人口计生卫生管理体制。实施“单独两孩”政策，启动优生健康促进计划，促进人口长期均衡发展。

　　繁荣文化体育事业。加强思想道德建设，大力弘扬社会主义核心价值观。完善公共文化服务体系，加快构建县、镇、社区三级文化中心网络，建设社区文化中心240个，新增农村文化礼堂150个。建成市文化艺术大楼。鼓励民间开办博物馆、图书馆等文化设施，促进公共文化服务多元化。加强刘伯温等历史名人研究和宣传。抓好非物质文化遗产传承。推进雁楠、廊桥、矾矿申遗。办好第三届中国越剧节。健全文化市场体系，支持温州文化“走出去”，促进文化产业规模化、集约化、专业化发展。深入实施全民健身计划，加快奥体中心等公共体育设施建设，办好省第五届少数民族运动会，促进群众体育、竞技体育和体育产业协调发展，创成省体育强市。

　　努力扩大就业、促进创业。做好高校毕业生、农村转移劳动力、退役士兵等重点群体的就业指导与帮助，全年新增城镇就业8.5万人。推进大学生创业示范基地、创业园建设，多渠道创造就业岗位。提高劳动纠纷预防和处置能力，构建和谐劳动关系。

　　加强社会保障和养老服务。健全社保扩面长效机制，推进城乡居民大病保险制度建设，年内实现制度全覆盖。完善社会救助制度，提高低保标准。推进养老多元化发展，加快多功能护理型养老机构建设，实现农村社区居家养老服务照料中心全覆盖。切实保障妇女、儿童和残疾人合法权益。发展红十字、慈善公益事业。深化绿色生态殡葬改革。

　　健全住房保障和供应体系。加快保障性安居工程建设，实施公共租赁住房保障新模式，全市开工各类保障性安居工程1.5万套、竣工3000套，新增公共租赁住房保障3000户。全面完成市区“安置提速”三年任务。完善房地产市场调控措施，把握好供地结构、节奏，促进房地产市场平稳健康发展。

　　加强社会治理能力建设。依托城市信息化资源，拓展“数字城管”应用领域，促进信息互通共享。加快构建基层网格管理“一张网”，突出抓好居住房屋出租管理和新居民服务管理。开展十大重点领域安全生产专项整治，坚决防止重特大安全事故发生。改革完善食品药品安全监管体制，整顿和规范食品药品市场秩序，保障群众饮食用药安全。推进气象现代化。加强应急救援体系和防灾减灾工作规范化建设，提高突发公共事件应急处置水平。依法加强宗教事务及宗教场所管理，保持宗教领域和谐稳定。做好新形势下的民族、外事、侨务及援藏、援疆等工作。支持工会、共青团、妇联等群团组织开展工作。

　　深化“平安大市”创建。完善社会治安防控体系，严厉打击和有效防范各类违法犯罪活动。健全社会稳定形势分析和重大决策社会稳定风险评估机制，着力化解社会矛盾。畅通群众诉求表达渠道，规范涉法涉诉信访工作，解决好信访热点和遗留问题。理顺社区矫正管理体制。健全司法救助和法律援助制度，完善人民调解、行政调解、司法调解联动工作体系。加强舆情引导管理，完善网络突发事件处置机制。

　　支持驻温解放军和武警部队建设，加强国防教育、国防动员和人民防空等工作，争创国家双拥模范城市“四连冠”。

　　各位代表！温州正处在负重攀登、爬坡过坎阶段，复杂的形势、严峻的挑战、繁重的任务，给政府工作提出了新的更高的要求。全体政府工作人员务必振奋精神、增强自信，不因眼前的困难而气馁，不为外界的议论而迷惘，坚定对“温州模式”的自信，主动发出“好声音”，积极释放“正能量”，凝心聚力推进改革发展。务必苦干实干、果敢担当，把改革发展的各项任务，一步一步展开、一件一件落实，一年一年进等升位。完善分级负责、层层落实的政府管理体制，各级政府、部门都要主动揽事、积极干事、认真办事。强化督查考核，实行“周协调、月公示、季点评、年考核”。健全新闻发言人制度，遇到重大事件、紧急情况，在第一时间启动响应、妥善处理、作出回应。建立鼓励创新、宽容失败、允许试错、责任豁免的机制，鼓励干部群众对照“四个有利于”大胆去试、放手去干。务必紧贴群众、改进作风，深入开展党的群众路线教育实践活动，坚决纠正“四风”，经常通过换位思考审视自身、改进不足，设身处地为基层、为企业、为群众排忧解难。大力弘扬雷厉风行、立说立行的工作作风，喊破嗓子不如甩开膀子，坐而论道不如干出实效，杜绝推诿扯皮，杜绝阳奉阴违，杜绝政策“出不了门、落不了地”。务必勤俭从政、干净干事，深入贯彻中央“八项规定”、省委“六个严禁”，严格执行公务接待、因公出国（境）、公车使用、办公用房等方面制度，市县两级政府“三公”经费预算支出削减30%。创新审计监督方式，提高财政资金使用绩效。加强效能监察和行政问责，深入开展“万人评议机关中层和基层站所”活动，严肃查处各种损害发展环境的不良行为，对阻扰企业发展、阻碍温商回归的人和事，一律从严处理。抓好反腐倡廉教育和廉政文化建设，加强对权力的监督制约，以零容忍态度坚决惩治腐败，营造风清气正的政务环境。

　　各位代表！依法行政是政府工作的基本准则。市政府将高度重视法治建设，深入实施法治政府建设考核评价体系，积极申报“较大的市”。认真落实市人大及其常委会各项决议决定，自觉接受人大依法监督和政协民主监督，认真办理人大代表建议和政协提案。积极听取民主党派、人民团体和各界人士的意见建议，主动接受司法监督、舆论监督和社会公众监督，恪尽职守，积极作为，不断提高执行力和公信力。

　　各位代表！赶超发展的使命激励我们奋蹄直追，全市人民的重托鞭策我们勇毅笃行。让我们紧密团结在以习近平同志为总书记的党中央周围，在中共温州市委的坚强领导下，团结和依靠全市人民，进一步解放思想、改革创新，真抓实干、砥砺奋进，为加快温州“赶超发展、再创辉煌”而努力奋斗！
